# VHL-dependent regulation of a β-dystroglycan glycoform and glycogene expression in renal cancer

**DOI:** 10.3892/ijo.2013.2066

**Published:** 2013-08-21

**Authors:** VASSILIS AGGELIS, RACHEL A. CRAVEN, JIANHE PENG, PATRICIA HARNDEN, LANA SCHAFFER, GILBERTO E. HERNANDEZ, STEVEN R. HEAD, EAMONN R. MAHER, ROBERT TONGE, PETER J. SELBY, ROSAMONDE E. BANKS

**Affiliations:** 1 Cancer Research UK Centre, Leeds Institute of Cancer and Pathology, St. James’s University Hospital, Leeds LS9 7TF, UK;; 2 The Scripps Research Institute, La Jolla, CA 92037, USA;; 3 Academic Department of Medical Genetics, Addenbrooke’s Treatment Centre, Cambridge CB2 0QQ;; 4 Waters Limited, Manchester M22 5PP, UK

**Keywords:** dystroglycan, glycosylation, renal cell carcinoma, shotgun proteomics, von Hippel-Lindau

## Abstract

Identification of novel biomarkers and targets in renal cell carcinoma (RCC) remains a priority and one cellular compartment that is a rich potential source of such molecules is the plasma membrane. A shotgun proteomic analysis of cell surface proteins enriched by cell surface biotinylation and avidin affinity chromatography was explored using the UMRC2- renal cancer cell line, which lacks von Hippel-Lindau (VHL) tumour suppressor gene function, to determine whether proteins of interest could be detected. Of the 814 proteins identified ∼22% were plasma membrane or membrane-associated, including several with known associations with cancer. This included β-dystroglycan, the transmembrane subunit of the *DAG1* gene product. VHL-dependent changes in the form of β-dystroglycan were detected in UMRC2−/+VHL transfectants. Deglycosylation experiments showed that this was due to differential sialylation. Analysis of normal kidney cortex and conventional RCC tissues showed that a similar change also occurred *in vivo*. Investigation of the expression of genes involved in glycosylation in UMRC2−/+VHL cells using a focussed microarray highlighted a number of enzymes involved in sialylation; upregulation of bifunctional UDP-N-acetylglucosamine 2-epimerase/N-acetylmannosamine kinase (GNE) was validated in UMRC2− cells compared with their +VHL counterparts and also found in conventional RCC tissue. These results implicate VHL in the regulation of glycosylation and raise interesting questions regarding the extent and importance of such changes in RCC.

## Introduction

Renal cell carcinoma (RCC) accounts for 3% of adult solid tumours and ∼30–40% of patients present with metastatic disease which has a poor prognosis, with a 5-year survival of <10%. Identification of novel therapeutic targets or biomarkers for prognostic, diagnostic or predictive use remains a priority. Cell surface proteins are involved in a number of vital cellular processes which are altered in tumourigenesis and constitute ideal targets for small molecule or antibody based therapies. Soluble shed forms may also act as circulating biomarkers, making this a subcellular compartment of particular interest in proteomic-based biomarker identification studies.

Investigation of the most common form of inherited RCC led to identification of the von Hippel-Lindau (VHL) tumour suppressor gene (1) and it is now clear that loss of VHL function also occurs in a large proportion of sporadic RCCs of the conventional (clear cell) subtype (2). VHL has been implicated in numerous biological processes and has a well established role in regulation of the transcription factor hypoxia-inducible factor (HIF), acting as the substrate recognition component of an E3 ubiquitin ligase complex that targets HIF-α subunits for polyubiquitination and proteasomal degradation in an oxygen-dependent manner. In cells exposed to hypoxia or lacking functional VHL, HIF-α is stabilised, resulting in a number of gene expression changes including the upregulation of vascular endothelial growth factor (VEGF), platelet-derived growth factor (PDGF) and carbonic anhydrase IX (CAIX). It is clear that although the HIF pathway is central to VHL function and tumourigenesis, HIF-independent functions, some of which involve other substrates of VHL ubiquitin ligase, contribute to its role (3,4).

Targeting cell surface proteins that are downstream of VHL is already being exploited, as illustrated by the receptor tyrosine kinase inhibitors sunitinib and sorafenib (5). Similarly CAIX, one of the most consistently upregulated proteins in conventional RCC, has been investigated in many studies including assessment of soluble forms in serum and urine (6). The plasma membrane was therefore chosen as a subcellular fraction to focus on for biomarker identification. During optimisation of a plasma membrane protein enrichment strategy based on cell surface biotinylation and avidin affinity chromatography for a comparative study (7) using the VHL-defective UMRC2- renal cancer cell line as a model system, purified proteins were catalogued using 1D PAGE followed by in-gel tryptic digestion and LC-MS/MS (GeLC-MS/MS). This identified several plasma membrane proteins previously associated with RCC and a number of proteins of interest as potential biomarkers due to their dysregulation in other cancers and/or their known cellular functions. These included β-dystroglycan, the transmembrane subunit of the dystroglycan 1 protein, which was selected for further study and shown to exhibit a VHL-dependent change in glycoform in UMRC2 cells. Using an oligonucelotide array targeting genes involved in glycosylation, changes in several key enzymes were found in UMRC2−/+VHL cells supporting a role for VHL-mediated changes in glycosylation in tumourigenesis. Altered expression of bifunctional UDP-N-acetylglucosamine 2-epimerase/N-acetylmannosamine kinase (GNE) was confirmed in UMRC2−/+VHL cells and also found to occur in conventional RCC.

## Materials and methods

### Cell culture and human tissue samples

VHL-deficient RCC cells (UMRC2, RCC4 and 786-0) transfected with vector control (−) and VHL (+VHL) were cultured as previously described (8). For validation studies, samples of macroscopically viable conventional RCC representing a range of grades (1–4) and stages (I–IV) of disease and matched distant normal renal cortical tissue were selected from a bank of fresh frozen samples collected and processed as previously described (8) following ethics committee approval and with informed consent.

### Preparation of plasma membrane fractions and whole cell lysates

A modified protocol of the method described by Zhao and co-workers (9) was adopted to isolate cell surface exposed plasma membrane proteins, using biotin-labelling of cells with EZ-link Sulfo-NHS-S-S-biotin (Perbio Science UK Limited, Cramlinghton, UK) and subsequent purification of biotinylated proteins with streptavidin sepharose™ high performance beads (GE Healthcare, Little Chalfont, UK). Each step of the protocol was optimised to maximise yield and enrichment of plasma membrane proteins; details of the final protocol have been described elsewhere (7). Whole tissue and cell lysates were prepared in RIPA buffer containing Complete™ mini protease inhibitor cocktail tablet (1 per 2.5 ml; Roche, Burgess Hill, UK) or Laemmli sample buffer as previously described (7).

### Western blotting

Western blotting was carried out using the Envision^TM+^-based detection system (Dako, Ely, UK) (8). Primary antibodies against the following proteins were used: rabbit polyclonal antibodies to GLUT-1 (Abcam plc, Cambridge, UK; 1:8,000), GNE (Sigma-Aldrich, Poole, UK; 1:250) mouse monoclonal antibodies to β-actin (Sigma-Aldrich, clone AC15, 1:400,000), β-dystroglycan (BD Biosciences, San Jose, CA, USA; clone 56, 0.5 *μ*g/ml), β-dystroglycan (Novocastra, Milton Keynes, UK; clone 43DAG/8D5, 0.1 *μ*g/ml), Golgin-84 (Abcam plc; clone 26, 0.5 *μ*g/ml), glucose regulated protein (GRP) 94 (Bioquote Limited, York, UK; clone 9610, 0.5 *μ*g/ ml), heat shock protein (HSP) 70 (Bioquote Limited; clone C92F3A-5, 0.05 *μ*g/ml), lamin A/C (BD Biosciences; clone 14, 0.75 *μ*g/ml), NADH ubiquinol oxidoreductase 39 kDa (Invitrogen; clone 20C11, 0.5 *μ*g/ml), Na/K-ATPase α1 (Novus Biologicals Inc., Littleton, USA; clone 464.4, 0.4 *μ*g/ ml). Western blots were normalised using parallel Coomassie-stained gels and additionally by probing with antibodies to β-actin. The optimal concentration of primary antibodies was pre-determined by titrations using whole UMRC2- cell lysates and linearity was confirmed by probing serial dilutions of protein load. Negative control blots were probed with irrelevant antibodies. Western blots were scanned as 12-bit images using a Personal Densitometer SI (GE Healthcare) and analysed using ImageQuant software.

### GeLC-MS/MS analysis of the enriched plasma membrane fraction

Purified plasma membrane proteins (40 *μ*g) from UMRC2- cells were resolved by SDS-PAGE (10% T). Gels were stained with colloidal Coomassie and lanes divided into 2-mm gel slices which were subjected to in-gel tryptic digestion as previously described (7). Online nano-LC/MS/ MS was performed on an Agilent 1100 nano-HPLC system (Agilent Technologies, South Queensferry, UK) coupled with a QSTAR XL (Applied Biosystems, Warrington, UK).

Protein Pilot (version 1.0, Applied Biosystems) and Analyst (version 2.0, Applied Biosystems) were used to extract and process the MS/MS spectra. Data were searched against the Celera mammalian protein database (KBM55.0.20050302. fasta) restricted to human (187835 entries) with the Paragon algorithm (10) using the following parameters: digestion: trypsin, search effort: rapid, Instrument: QSTAR ESI (mass tolerance 0.2 Da for MS and MS/MS ions), cysteine alkylation: iodoacetamide. Protein identification required at least two peptides with 95% confidence. The ProGroup algorithm was used to generate a minimal set of protein identifications. False discovery rates at the protein level (that is, requiring two significant peptides) were estimated to be 0.0024% by searching a decoy version of the database generated by EMBOSS (11).

### Protein deglycosylation of whole cell lysates

Removal of N-linked glycans from glycoproteins was carried out using the GlycoProfile™ II Enzymatic In-solution N-Deglycosylation kit (Sigma-Aldrich), according to the manufacturer’s protocol. Briefly, protein from whole UMRC2−/+ cell extracts prepared in RIPA buffer was adjusted to 1X reaction buffer (20 mM NH_4_HCO_3_) and 2 *μ*l of denaturing solution [2% (w/v) OCG, 100 mM β-mercaptoethanol] was added and samples incubated for 10 min at room temperature. Deglycosylation was carried out for 17 h at 37°C with the addition of 10 *μ*l of peptide-N-glycosidase (PNGase) F (500 U/ml). The reaction was terminated by freezing the samples at −80°C. Complete removal of N- and O-linked glycans was performed using the Glycoprotein Deglycosylation kit (Merck, Nottingham, UK), according to the manufacturer’s protocol. Protein from whole UMRC2−/+VHL cell extracts prepared in RIPA buffer was diluted in 5X reaction buffer (250 mM sodium phosphate buffer, pH 7.0), followed by the addition of 2.5 *μ*l of denaturing solution [2% (w/v) SDS, 1 M β-mercaptoethanol] and 3.75 *μ*l of 15% (v/v) Triton X-100. Enzymatic deglycosylation was carried out by the addition of 1 *μ*l of each enzyme (Table I) and samples were incubated at 37°C for 24 h. The reaction was terminated by freezing the samples at −80°C. In both cases mock reactions where deglycosylation enzymes were substituted with an equivalent volume of H_2_O were carried out in parallel.

Deglycosylation was examined by Western blotting with analysis of GLUT-1 being used as an internal control. In addition, as an independent deglycosylation control, bovine fetuin (Sigma-Aldrich or provided in the Glycoprotein Deglycosylation kit) was deglycosylated under the same conditions as the cell lysates and the glycan removal and subsequent shift in molecular weight was observed by silver staining.

### Glycoarray analysis

RNA was extracted from three independent replicates of each of the UMRC2, RCC4 and 786-0 cell lines (all −/+VHL) using the Qiagen RNeasy Mini kit and used to probe the GLYCOv3 oligonucleotide array (https://www.functionalglycomics.org), a custom Affymetrix GeneChip (Affymetrix, Santa Clara, CA, USA) designed for the Consortium for Functional Glycomics and including probes for 1,188 human probe-ids encoding a number of classes of protein including glycosyltransferases, glycan degradation proteins, nucleotide sugar synthesis and transporter proteins and glycan binding proteins (https://www.functionalglycomics.org). Total RNA sample quality was checked with an Agilent Bioanalyzer (Agilent Technologies, Palo Alto, CA, USA). RNA from each preparation was labelled using the MessageAmp II-Biotin Enhanced Amplification kit (Ambion Inc., Austin, TX, USA). Hybridization and scanning of the GLYCOv3 chip were performed according to the Affymetrix recommended protocols (12). The chips were scanned using the Affymetrix GeneChip Scanner 3000 using default settings and a target intensity of 250 for scaling. Chips had a background <100 intensity units and a GAPDH 3′/5′ ratio <1.8. Robust Multichip Average (RMA) was used to convert the intensity values to expression values (13,14). RMA consists of a three step approach which uses a background correction, a quantile normalization and summarizes the probe set information by using Tukey’s median polish algorithm. All processing of the data was performed within the Bioconductor project and the R program software (R is available as Free Software under the terms of the Free Software Foundation’s GNU General Public License). The-fold changes and standard errors were estimated by fitting a linear model for each gene and empirical Bayes smoothing was applied to the standard errors for all the samples at the same time. The linear modeling approach and the empirical Bayes statistics as implemented in the Limma package in the R software were employed for differential expression analysis (15,16). Statistics were obtained for transcripts with the multiple testing adjusted (Benjamini-Hochberg) p-value level of 0.05. Filtering was performed so that probe-sets with a fold change of <1.3 were eliminated from the results.

## Results

**Figure 1.**
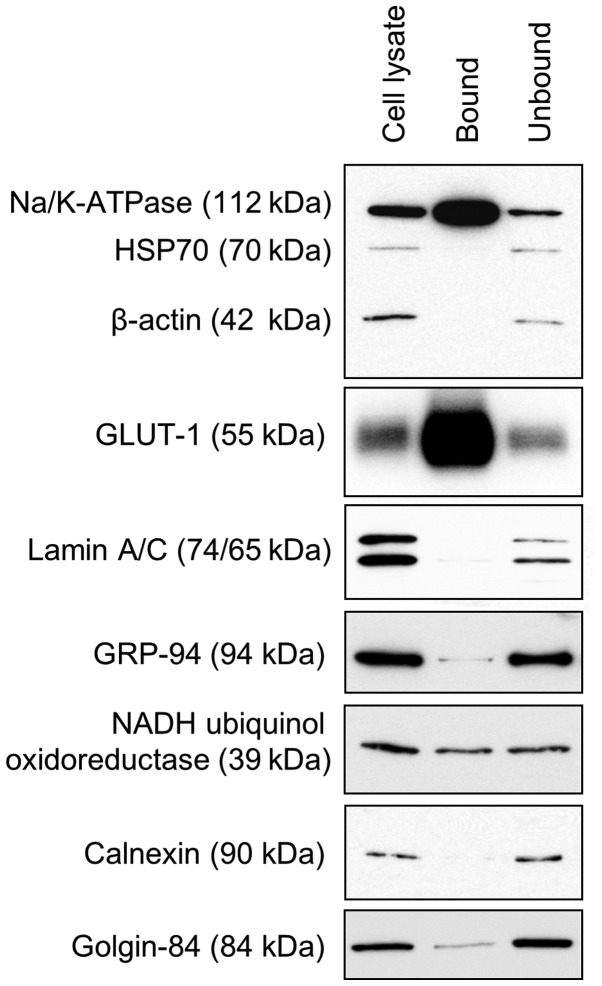
Enrichment of plasma membrane proteins by cell-surface biotinylation and avidin affinity chromatography. Plasma membrane proteins were enriched from UMRC2− cells by cell surface biotinylation and avidin-affinity chromatography. The extent of enrichment was evaluated by comparing the plasma membrane protein (bound) fraction with the intracellular (unbound) fraction and a whole cell lysate. Protein (5 *μ*g) was separated by SDS-PAGE and analysed by immunoblotting with antibodies specific to distinct subcellular marker proteins: Na/K ATPase α1 and GLUT-1 (plasma membrane), HSP70 (cytoplasm), β-actin (cytoplasm/cytoskeleton), Lamin A/C (nucleus), GRP94 (ER lumen), Calnexin (ER membrane), Golgin-84 (Golgi) and NADH ubiquinol oxidoreductase 39 kDa (mitochondria).

A method for enrichment of plasma membrane proteins using cell surface biotinylation and avidin affinity chromatography was optimised using the VHL-defective renal cancer cell line UMRC2-. Western blot analysis (Fig. 1) showed significant enrichment of the plasma membrane proteins Na/K-ATPase α1 and GLUT-1 compared to the unbound fraction or a whole cell lysate. The abundant cytosolic proteins HSP70 and β-actin were almost undetectable as were proteins specific to endoplasmic reticulum (GRP94 and calnexin) and the nucleus (lamin A/C). Low levels of golgin-84 were found whilst the mitochondrial protein NADH-ubiquinol oxidoreductase 39 kDa was present at more significant levels, indicating that some contamination may be present.

Using a GeLC-MS/MS approach, a total of 3,991 peptides were identified corresponding to 814 unique proteins with at least two significant peptides (the complete date set is available at www.proteomics.leeds.ac.uk), of which 183 (22%) were known plasma membrane proteins (a selection of these are shown in Table II); this represents a significant enrichment compared with 5% of the 956 proteins identified in a parallel analysis of a whole cell lysate. The identified proteins included several of interest in the context of VHL/RCC including integrin α3, transferrin receptor 1, epidermal growth factor receptor (EGFR) and CAIX.

One protein selected for further study was the *DAG1* gene product dystroglycan-1, a protein that has been previously implicated in carcinogenesis (17). Dystroglycan-1 comprises two subunits, α and β, which are generated by proteolytic cleavage of the α/β precursor polypeptide. The peptides identified in this study (corresponding to amino acids 702–714, 783–793 and 795–823) were all from β-dystroglycan, a 43-kDa type I transmembrane protein consisting of amino acids 654–895 of the molecule (18) that anchors the extracellular α-subunit to the cell surface.

**Figure 2.**
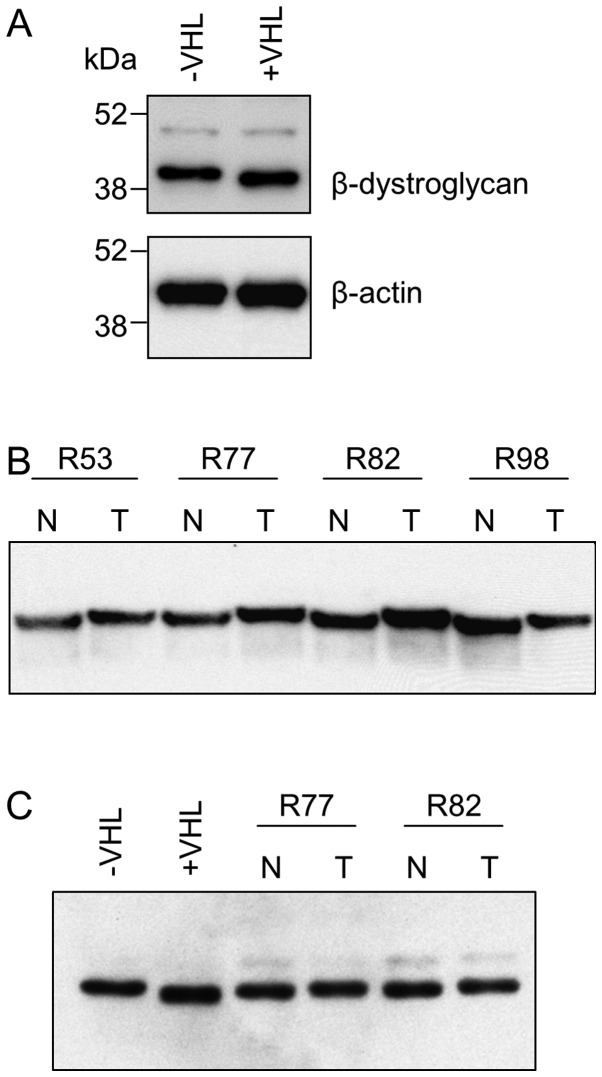
Different forms of β-dystroglycan are expressed in UMRC2−/+VHL cells and in tumour/normal kidney tissues. (A) Whole cell extracts (5 *μ*g) were prepared from UMRC2−/+VHL cells and analysed by immunoblotting with antibodies to β-dystroglycan. β-actin was used as a control. (B) Whole tissue lysates were prepared from patient-matched normal kidney cortex (N) and clear cell RCC (T). Protein (10 *μ*g) was resolved by SDS-PAGE and analysed by immunoblotting with antibodies to β-dystroglycan. (C) Extracts prepared from UMRC2−/+VHL cells and patient matched normal kidney cortex (N) and clear cell RCC (T) tissue were analysed by immunoblotting with antibodies to β-dystroglycan.

Comparative analysis of UMRC2−/+VHL whole cell lysates by Western blotting using an antibody raised against amino acids 655–767 (clone 56) towards the N-terminus of the β-dystroglycan molecule that recognises the full length protein (43 kDa) but not the fragment reported to migrate at ∼31 kDa (19,20) showed no difference in expression level but a slightly increased electrophoretic mobility (corresponding to a difference of <5 kDa) in UMRC2+VHL cells (Fig. 2A). This was confirmed using an alternative anti-β-dystroglycan antibody (clone 43DAG/8D5) recognising a C-terminal epitope. This alteration was not seen in two other renal cancer cell lines (RCC4 and 786-0 −/+ VHL) but a similar change was seen *in vivo* in 12/15 matched normal and RCC tissue samples (Fig. 2B). The form of β-dystroglycan seen in tumour tissue was found to co-migrate with that in UMRC2− cells but the form in normal renal tissue migrated more slowly than that in UMRC2+VHL cells, thus the overall difference was smaller in magnitude (Fig. 2C).

**Figure 3.**
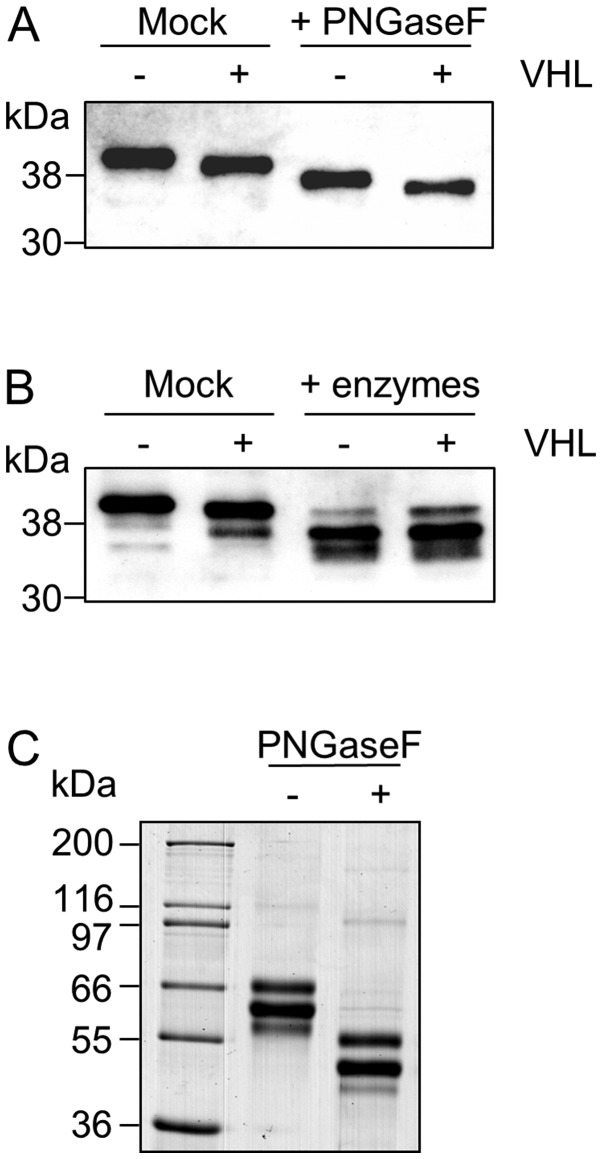
Different forms of β-dystroglycan arise due to differential glycosylation. UMRC2−/+VHL lysates were deglycosylated (A) using PNGase F to remove N-linked glycans and (B) using a cocktail of enzymes to remove N- and O-linked glycans (for details of these enzymes see Table I). Mock digests were carried out without enzyme(s). Changes in the gel mobility of β-dystroglycan were measured using immunoblotting. (C) Deglycosylation of bovine fetuin (as measured by SDS PAGE and silver staining) was monitored as an external control; a representative example is shown.

**Figure 4.**
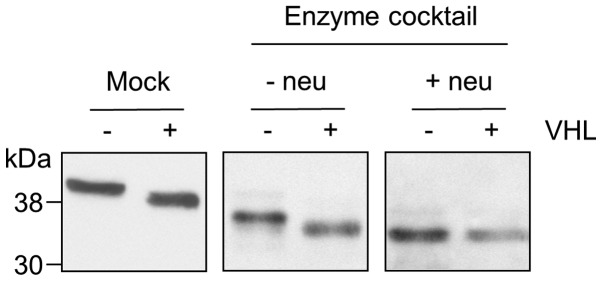
Different forms of β-dystroglycan arise due to differential sialylation. UMRC2−/+VHL lysates were deglycosylated using a cocktail of enzymes to remove N- and O-linked glycans. Reactions omitting (− neu) or including (+ neu) α2–3,6,8,9-neuraminidase were compared with mock digests carried out without enzymes using immunoblotting.

Removal of N-glycans with PNGase F resulted in a shift in molecular weight of both β-dystroglycan isoforms with the difference in size between the UMRC2− and +VHL cells still apparent (Fig. 3A). Using a combination of exoglycosidases together with PNGase F to remove both N- and O-linked glycans eliminated the difference in size seen between UMRC2- and +VHL cells (Fig. 3B) strongly supporting differential glycosylation as being the cause of the difference, with O-linked glycans contributing at least part of the change. For deglycosylation experiments, GLUT-1 was monitored as an internal control and bovine fetuin as an external control (for an example see Fig. 3C). When α2–3,6,8,9-neuraminidase, which specifically removes all non-reducing terminal branched and unbranched sialic acid residues, was omitted from the deglycosylation reaction, the difference in size of β-dystroglycan between UMRC2− and +VHL cells was still apparent (Fig. 4). Conversely, if deglycosylation was carried out using only α2–3,6,8,9-neuraminidase, the difference in size was eliminated (data not shown). Taken together these data indicate that the difference between β-dystroglycan isoforms is due predominantly to a change in the level of sialylation. The presence of protease inhibitors in the extracts used for the experiment, together with the absence of a change in mobility in the mock reactions carried out without enzymes strongly suggest that proteolytic activity did not contribute to the reactions (additional bands were present in both the mock reactions and the deglycosylated samples in the example shown in Fig. 3B, but these were of significantly lower intensity than the major forms and not present in every reaction as illustrated in Fig. 4). The smaller magnitude of change in tissue made resolution of forms of β-dystroglycan more difficult thus confident interpretation of deglycosylation experiments with tissues was not possible.

Examination of the expression profiles of genes involved in glycosylation using a glycosylation focussed microarray showed changes in several molecules (the complete date set is available at www.proteomics.leeds.ac.uk), several of which related to possible alterations in sialylation (Table III). These included GNE, which encodes bifunctional UDP-N-acetylglucosamine 2-epimerase/N-acetylmannosamine kinase, which was upregulated in UMRC2− cells and NPL, which encodes a sialic acid lyase, which was downregulated. As a positive control, changes in other gene classes on the chip which were found in UMRC2 cells included upregulation of TGF-β and downregulation of clusterin in -VHL cells which are both known VHL-associated changes (21,22).

**Figure 5.**
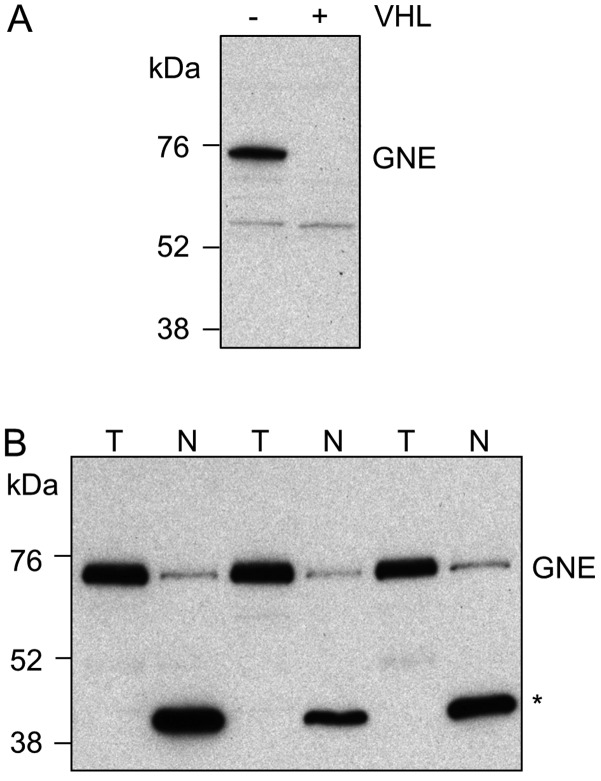
Altered expression of GNE in UMRC2−/+VHL cells and in RCC tissue. Protein (5 *μ*g) from (A) UMRC2−/+VHL cells and (B) patient matched normal kidney cortex (N) and RCC tissue (T) was separated by SDS-PAGE and analysed by immunoblotting with antibodies specific to GNE. (^*^) indicates a lower molecular weight band of unknown identity seen exclusively in normal kidney tissue.

Expression of GNE, the gene of interest which showed the greatest magnitude of change at the mRNA level between UMRC2−/+VHL cells, was investigated further. Western blot analysis showed that GNE protein levels changed in UMRC2−/+VHL cells in a manner that paralleled the changes seen at the mRNA level (Fig. 5A). Furthermore, in matched normal kidney cortex and conventional RCC tissues, GNE was found to be upregulated in 6/8 tumours (Fig. 5B). In normal kidney tissue a dominant lower molecular weight band was also seen; unlike the higher molecular weight band, this was not recognised by an alternative antibody to GNE raised against a different epitope and it remains to be determined whether this represents a smaller form of GNE or a cross reacting protein species.

## Discussion

The tumour suppressor gene *VHL* plays a central role in development of conventional RCC and the characterisation of *VHL*-regulated proteins and pathways offers promise in identifying new biomarkers and therapeutic targets. In RCC both global mRNA expression profiling of VHL cell line pairs (23–25) and complementary proteomic approaches (7,8,22,26) have succeeded in identifying changes that are relevant in tumourigenesis.

Previous analysis of the membrane proteome of renal cancer cells fractionated from a post-nuclear supernatant using a 60% sucrose cushion followed by a 15–60% sucrose gradient identified high expression of CD70 and showed that this protein could act as a target in antibody-targeted cytotoxic therapy (27). A further study used cell surface capturing (CSC) technology, where glycans are labelled with biocytin hydrazide and following digestion, labelled N-glycopeptides purified by avidin affinity chromatography, combined with stable isotope labelling with amino acids in cell culture (SILAC) to compare the cell surface of −/+VHL cells (26). In the present study, cell surface biotinylation and avidin affinity chromatography was used to enrich plasma membrane proteins as part of method development for a quantitative comparative proteomic study. The presence of NADH ubiquinol oxidoreductase 39 kDa in the enriched fraction suggested some contamination from other organelles, but this protein is a subunit of a respiratory chain complex demonstrated to localise to the plasma membrane (28). Similarly, a number of proteomic studies have reported intracellular proteins on the cell surface, such as cytoplasmic and ER lumenal chaperones (29–31). The enrichment strategy described here was used in combination with SILAC in a study which identified upregulation of the adhesion molecules CD166 and CD147 in VHL defective cells and some RCC tissues (7).

Whilst examining the enriched fraction to assess the extent of profiling of proteins of relevance in RCC, β-dystroglycan was selected for further analysis due to the known involvement of dystroglycan in cancer. Dystroglycan is a transmembrane glycoprotein encoded by the *DAG1* gene that is processed into two subunits − the transmembrane β domain and the extracellular α domain. Loss of α-dystroglycan expression and correlations with prognosis have been reported in a number of tumour types (32–36). In RCC, loss of α-dystroglycan correlated with high grade disease and was an independent predictor of shorter disease-free and overall survival (37). Combined loss of α-dystroglycan and p27^kip1^ defined a group of patients with particularly poor outcome (38). Many studies analysing α-dystroglycan used antibodies recognising glycosylation-dependent epitopes and changes in glycosylation have been suggested to account for loss of α-dystroglycan staining (39,40). Changes in expression of both LARGE and β3-N-acetylglucosaminyltransferase-1 restored glycosylation of α-dystroglycan and altered tumour cell behaviour (41,42).

Changes in expression of β-dystroglycan in cancer are less consistent with some studies finding no change in expression. Loss of β-dystroglycan was found in some cancers including prostate, breast, colon and oesophageal (32,43–45) and relationships with progression were reported for breast and colon cancers (32). In oral SCC loss of β-dystroglycan was reported in poorly differentiated tumours (19) whilst in a separate study the presence of the 31 kDa β-dystroglycan fragment correlated with lymph node metastasis and tumour differentiation (46).

Characterisation of β-dystroglycan showed that its form changed in UMRC2 cells in a VHL-dependent manner. No evidence was found to suggest that this was due to changes in phosphorylation or alternative splicing (using dephosphorylation with lambda protein phosphatase and by RT-PCR respectively, data not shown). However, deglycosylation experiments indicated that this change was due, at least in part, to differential sialylation. A similar but smaller change was also seen in the majority of RCC samples compared with matched normal kidney cortex. Previous studies in RCC did not report this change in glycosylation in β-dystroglycan (38) which may be due to the gel systems used as resolution of the forms, which differ by <5 kDa, is difficult, especially in tissues.

As mentioned above, a truncated ∼31-kDa fragment of β-dystroglycan lacking the extracellular domain has been identified in cell lines and tissues (20,47) with processing by matrix metalloproteinases being implicated in its formation (46–48); an MMP-9 cleavage site has recently been defined (49). Tyrosine phosphorylated forms have also been described (50,51). In a study examining the role of dystroglycan in prostate cancer cell lines, β-dystroglycan was shown to exhibit reversible cell density dependent changes in form, with lower molecular weight forms of 38–43 kDa due to mis-glycosylation and bands at 31 and 26 kDa resulting from proteolysis being seen in supra-confluent cells (52). The altered glycosylation in our study seems to be distinct from this effect. Treatment with PNGase F resulted in a similar increase in gel mobility of β-dystroglycan in UMRC2− and +VHL cells thus N-glycosylation was present irrespective of VHL status. The cells used in our study were all harvested at the same growth state (that is, approaching confluence), but it is possible that VHL alters the point at which a density-dependent change in form is induced.

Possible mechanisms underlying the VHL-dependent changes in sialylation were investigated using a glycoarray. Overall, the glycoarray results for the three −/+VHL cell line pairs analysed did not show obvious patterns, which may reflect the complexity of glycosylation, the potential for differences between differing genetic backgrounds and the limitations of cell line models. Altered expression of glycogenes involved in sialylation that were seen in UMRC2 cells, like the change in form of β-dystroglycan, were restricted to this cell line, with the exception of NPL, expression of which was found to be VHL-dependent in all three cell lines. Previous studies have also found differences in VHL-dependent gene expression in different cell lines and this is apparent here not just for genes involved in glycosylation but also known VHL-regulated genes present on the array, with no one cell line pair behaving as an outlier.

In UMRC2−/+VHL cells, lower expression of NEU1 which encodes sialidase-1, which localises to the lysosome and the cell surface and increased expression of the sialyltransferases ST3GAL6 and ST6GAL1, in UMRC2− cells may contribute to altered sialylation. Similarly, upregulation of GNE, which is a key enzyme in sialic acid biosynthesis, together with downregulation of NPL, which is involved in sialic acid turnover, in UMRC2− cells may alter the availability of sialic acid and thereby affect sialylation. Indeed there is mounting evidence in the literature that substrate availability and levels of GNE do impact on sialylation. In an analysis of N-linked sialoglycopeptides in human pancreatic carcinoma (SW1990) cells, increased metabolic flux through the sialic acid pathway by exogenously supplied substrate was found to selectively increase the sialylation of individual glycoproteins (53). In hematopoietic cell lines, GNE was found to be an important regulator of cell surface sialylation (54) and knockdown of GNE in HEK293 cells reduced total cell surface sialic acid content (55). Expression of GNE in UMRC2−/+VHL cells validated the changes found using the microarray, with reduced expression of GNE being seen in VHL transfectants and GNE levels were also upregulated in a significant proportion of tumours compared to normal renal tissue. Changes in tumour tissue at the mRNA level reported in microarray data sets correlate with this result (56,57).

A similar finding of involvement of a tumour suppressor gene in glycosylation has been previously reported, with altered expression of glycosyltransferases and decreased sialylation of N- and O-glycans being seen in Capan-1 pancreatic carcinoma cells following transfection with p16^INK4a^ (58). In an extension of this study, decreased levels of GNE were found to be an important consequence of p16^INK4a^ transfection, correlating with loss of membrane bound sialic acid and hyposialylation of α5 and β1 integrin (56). The changes in these integrin subunits were very similar to the change seen in β-dystroglycan.

The results described here raise the question of the functional impact of changes in glycosylation of β-dystroglycan in renal cancer and there is clearly enormous potential for glycosylation-dependent biomarkers (59). Alterations in glycosylation associated with cancer progression are well documented and include changes in sialylation (reviewed in ref. 60). Changes in glycosylation in RCC have been shown using lectin staining (61) and we have previously described VHL-dependent changes in glycosylation of CD166 (7). The extent to which glycosylation of other proteins can also be regulated by VHL remains to be determined. Further work is now required to build on the preliminary findings presented here and establish the extent of changes in glycosylation in RCC that may be attributable to VHL and their functional consequences.

## Figures and Tables

**Table I.** Exoglycosidases used to remove N- and O-linked glycans from proteins.

Enzyme	Substrate
N-Glycosidase F (5,000 U/ml)	All asparagine-linked complex, hybrid, or high mannose oligosaccharides unless α1,3-core fucosylated
Endo-α-N-acetylgalactosaminidase (1.25 U/ml)	Serine- or threonine-linked unsubstituted Galβ1,3GalNAcα
α2–3,6,8,9-neuraminidase (5 U/ml)	Non-reducing terminal branched and unbranched sialic acids
β-1,4-galactosidase (3 U/ml)	Only β1,4-linked, non-reducing terminal galactose
β-N-acetylglucosaminidase (45 U/ml)	All non-reducing terminal β-linked N-acetylglucosamine residues

**Table II.** Selected proteins identified by GeLC-MS/MS.

Accession no.	Protein name	Unused protein score	Percent coverage	Significant peptides (>95%)
Q16790	Carbonic anhydrase IX	10.00	20.92	5
O43570	Carbonic anhydrase XII	6.00	20.06	3
P00533	Epidermal growth factor receptor	53.81	42.81	25
P08183	Multidrug resistance protein 1	11.04	19.14	3
Q969J9	Dystroglycan 1	5.40	7.26	3
Q13740	MEMD protein (CD166)	24.71	41.24	12
P02786	Transferrin receptor protein 1	47.05	52.24	23
Q8WUM6	Integrin β-1	30.64	38.47	14
P05106	Integrin β-3	12.07	13.83	6
P06756	Integrin α-V	50.17	46.95	21
P18084	Integrin β-5 precursor	16.30	25.28	7
P23229	Integrin α-6 precursor	4.53	3.19	2
P26006	Integrin α-3	19.99	18.29	9
P21796	Voltage-dependent anion-selective channel protein 1	15.46	50.00	7
P45880	Voltage-dependent anion-selective channel protein 2	11.05	37.76	5
Q9Y277	Voltage-dependent anion-selective channel protein 3	16.16	54.77	8
P05023	Sodium/potassium-transporting ATPase α-1 chain	54.27	40.66	25
P54709	Sodium/potassium-transporting ATPase β-3 chain	4.18	23.30	2
O15153	Sodium bicarbonate cotransporter	30.25	34.40	13
P53985	Monocarboxylate transporter 1	11.82	14.80	6
P13987	CD59	3.99	35.94	2
P55285	K-cadherin	6.19	9.62	3
P19022	N-cadherin	9.64	14.68	4
Q6PHR3	Melanoma cell adhesion molecule	23.42	37.31	12
P27487	Dipeptidyl peptidase IV	14.37	22.19	7

Details of the peptides are available at www.proteomics.leeds.ac.uk.

**Table III.** Genes involved in sialylation with altered expression in UMRC2−/+VHL cells.

Gene name	Accession no. (NCBI)	Protein name	Fold change in UMRC2^a^
NPL	AF338436	Sialic acid lyase	1.8-fold ↓
GNE	NM_005476.2	UDP-GlcNAc-2-epimerase/ManAc kinase	5.2-fold ↑
ST3GAL6	NM_006100.2	Sialyl transferase 10	2.1-fold ↑
ST6GAL1	NM_173216.1	Sialyltransferase 1	1.7-fold ↑
NEU1	BC000722	Sialidase-1	1.3-fold ↓

a For all changes p<0.01. The complete data set is available at www.proteomics.leeds.ac.uk and details of the glycoarray can be accessed online at www.functionalglycomics.org.
